# Management of hair loss after severe acute respiratory syndrome coronavirus 2 infection: Insight into the pathophysiology with implication for better management

**DOI:** 10.1111/1346-8138.16475

**Published:** 2022-05-27

**Authors:** Manabu Ohyama, Kiichi Matsudo, Toru Fujita

**Affiliations:** ^1^ Department of Dermatology Kyorin University Faculty of Medicine Tokyo Japan; ^2^ Clinical Development, Research and Development Headquarters Self‐Medication Taisho Pharmaceutical Co.Ltd Tokyo Japan

**Keywords:** alopecia, COVID‐19, minoxidil, pandemic, quarantine

## Abstract

Coronavirus disease 2019 (COVID‐19) caused by severe acute respiratory syndrome coronavirus 2 (SARS‐CoV‐2) was declared a pandemic by the World Health Organization, and COVID‐19 continues to have a major impact on society. Numerous studies have reported impaired health sequelae after COVID‐19 recovery, one of which is hair loss. Individuals with hair loss experience a substantial mental burden, which potentially hinders their social life. However, few studies have systematically analyzed the details including hair loss. Therefore, we conducted a narrative review using PubMed on the frequency, associated comorbidities, disease characteristics, and treatment of hair loss after SARS‐CoV‐2 infection (HLASCI). Two search strings were used to identify 28 articles. Of note, most of the literature identified on COVID‐19 sequelae reported an emergence/occurrence of hair loss. HLASCI is speculated to be composed of a heterogeneous population, with the onset or exacerbation of telogen effluvium (TE), anagen effluvium, androgenetic alopecia (AGA), and alopecia areata (AA) reported as possible underlying mechanisms. Among these, acute TE is thought to be the primary cause of HLASCI, with COVID‐19 treatment and TE improvement being considered crucial for HLASCI management. An association between COVID‐19 and AA exacerbation has also been implicated with still insufficient evidence. Spontaneous recovery of TE can be expected once infection reduces; however, faster improvement in symptoms is expected to reduce the mental and social burden of patients. An additional search string identified 11 articles about TE treatment which suggested that the use of minoxidil may be beneficial. Topical minoxidil has been widely used for AGA patients, who have been speculated to exhibit poor resistance to SARS‐CoV‐2. Topical minoxidil may provide relief from HLASCI, but future clinical research is warranted to confirm this observation.

## 1 INTRODUCTION

A meta‐analysis of COVID‐19‐infected patients showed that 80% of patients developed one or more long‐term non‐respiratory symptoms with sequelae lasting weeks to months after resolution of the primary symptoms.^1^ This condition has been referred to as “Long COVID‐19,” with patients with this condition being called “Long Haulers.”^1^ Of note, one study reported hair loss as a sequela in 25% of patients.^1^ The reported frequency of hair loss as a sequela in other studies varies from 6% to 28.6%.^1^, ^2^, ^3^ Pre‐existing alopecia has been reported to be a risk factor for COVID‐19. Worsening of autoimmune alopecia such as alopecia areata (AA) as a complication of COVID‐19 has also been reported.^4^, ^5^, ^6^

Although hair loss after SARS‐CoV‐2 infection (HLASCI) has attracted global interest as a sequela of COVID‐19, its etiopathogenesis and pathophysiology have not been investigated in depth. Therefore, we conducted a narrative literature review to clarify the characteristics of HLASCI, especially focusing on its pathomechanism, and propose a probable therapeutic option.

## 2 METHODS

Articles published between January 2020 and September 2021, when the COVID‐19 pandemic started, in the PubMed database were included.

We used three search strings (1, 2, and 3) to identify studies reporting the disease state of hair loss with COVID‐19 and possible therapies.

### 2.1 [Search string 1]

((((SARS‐CoV‐2[Title/Abstract] AND (2020/1/1:2021/9/1[pdat])) OR (COVID[Title/Abstract] AND (2020/1/1:2021/9/1[pdat]))) OR (COVID19[Title/Abstract] AND (2020/1/1:2021/9/1[pdat]))) OR (COVID‐19[Title/Abstract] AND (2020/1/1:2021/9/1[pdat])) AND (2020/1/1:2021/9/1[pdat])) AND (((HAIRLOSS[Title/Abstract] AND (2020/1/1:2021/9/1[pdat])) OR (HAIR LOSS[Title/Abstract] AND (2020/1/1:2021/9/1[pdat]))) OR (ALOPECIA[Title/Abstract] AND (2020/1/1:2021/9/1[pdat])) AND (2020/1/1:2021/9/1[pdat])).

Telogen effluvium (TE) is a common cause of hair loss, which has been related to severe infection,^7^ and the aforementioned literature search suggested that HLASCI mainly comprised acute TE. Next, the relationship between acute TE and therapy was examined by search formulae 2 and 3. To maximize the range of detection, the words, “telogen” and “effluvium,” were separately adopted in Search string 2, while, in Search string 3, “telogen effluvium” was adopted as a single term to minimize the noise in the identification of treatments specific to the condition. From the viewpoint of the content and quality of articles, the search formula and inclusion/exclusion criteria for the articles to be reviewed were determined as follows:

### 2.2 [Search string 2]

((((SARS‐CoV‐2[Title/Abstract] AND (2020/1/1:2021/9/1[pdat])) OR (COVID[Title/Abstract] AND (2020/1/1:2021/9/1[pdat]))) OR (COVID19[Title/Abstract] AND (2020/1/1:2021/9/1[pdat]))) OR (COVID‐19[Title/Abstract] AND (2020/1/1:2021/9/1[pdat])) AND (2020/1/1:2021/9/1[pdat])) AND ((TELOGEN[Title/Abstract] AND (2020/1/1:2021/9/1[pdat])) OR (EFFLUVIUM[Title/Abstract] AND (2020/1/1:2021/9/1[pdat])) AND (2020/1/1:2021/9/1[pdat])).

### 2.3 [Search string 3]

(((treatment AND (2020/1/1:2021/9/1[pdat])) OR (treatment management AND (2020/1/1:2021/9/1[pdat])) AND (2020/1/1:2021/9/1[pdat])) OR ((((Therapeutic Use[MeSH Subheading] AND (2020/1/1:2021/9/1[pdat])) OR (Drug Therapy[MeSH Subheading] AND (2020/1/1:2021/9/1[pdat]))) OR (Drug Effects[MeSH Subheading] AND (2020/1/1:2021/9/1[pdat])) AND (2020/1/1:2021/9/1[pdat])) AND (Humans[MeSH Terms] AND (2020/1/1:2021/9/1[pdat])) AND (2020/1/1:2021/9/1[pdat])) AND (2020/1/1:2021/9/1[pdat])) AND (telogen effluvium[Title/Abstract] AND (2020/1/1:2021/9/1[pdat])).

### 2.4 [Inclusion and exclusion criteria]

Systematic reviews, literature reviews, case series reports, original articles, clinical trials, and observational studies were included in the search, and non‐English‐language articles, letters to the editor, commentaries, editorials, individual case reports, mini or brief reviews, pre‐clinical (animal/in vitro) studies, and non‐hair loss‐related articles were excluded.

## 3 RESULTS OF THE LITERATURE REVIEW

Table 1 shows the results of the literature search using the three search strings.

**TABLE 1:** Literature search results

Search formula	Initial hits	First pass inclusion^a^	Second pass inclusion^b^	Full data extracted^c^
①	84	66	35	21
②	25	22	9	7
③	41	33	24	11
Total	150	121	68	39

^a^ First pass review was based on a review of the title and abstract for relevance.

^b^ Second pass review was also conducted on the abstract and articles were included/excluded on the basis of the criteria.

^**c**^ Full‐text review further excluded articles on the basis of the exclusion criteria, but exclusion could only be determined with the full paper.

Using search strings 1, 2, and 3 in PubMed, we identified 84, 25, and 41 relevant articles for respective strings. On reviewing the abstracts and contents with additional three rounds of narrowing down the relevant articles according to the above‐mentioned inclusion and exclusion criteria; 21, 7, and 11 articles, respectively, were finally selected for each category.

The reasons for exclusion of full data extracted were letter to the editor, review article, no hair loss reported, hypothesis review article, case series, correspondence; does not meet inclusion criteria, only one patient met the criteria of hair loss and COVID‐19, commentary, infographics, single case report and in vitro study.

### 3.1 association between covid‐19 and hair loss

After full‐text review of the 21 articles on HLASCI were obtained. Table 2 summarizes the main types of hair loss that are mainly described.

**TABLE 2:** Summary of 21 articles extracted by string 1 and string 2

Article	Hair loss type	Summary of reports
1. Lopez‐Leon S, et al. *Res Sq*. 2021 Mar 1; doi:10.1101/2021.01.27.21250617.^1^	Hair loss (type not mentioned)	As per the systematic review and meta‐analysis, 80% of patients developed one or more long‐term symptoms. Hair loss occurred in 25%. Alopecia occurred in 178 of 658 patients
2. Cheng D, et al. *BMJ Open Respir Res*. 2021;8(1).^2^	Hair loss (type not mentioned)	In the cohort analyses of 1946 patients with COVID‐19, ongoing symptoms, including hair loss, were reported in 70% of survivors
3. Xiong Q, et al. *Clin Microbiol Infect*. 2021 Jan;27(1):89–95.^3^	Alopecia (type not mentioned)	A telephonic survey of 538 COVID‐19 survivors identified sequelae, including alopecia (*n* = 154, 28.6%) 3 months after discharge from hospital
4. Rinaldi F, et al. *Dermatol Ther (Heidelb)*. 2021;11(2):339–45.^4^	AA	A questionnaire‐based survey of 392 subjects indicated that 44% of subjects relapsed with AA after about 2 months from COVID‐19 infection
5. Kutlu Ö, Metin A. *Dermatol Ther*. 2020;33(6).e14096.^5^	AA, TE	Dermatology outpatient visits reduced from 2442 in 2019 to 738 in 2020 due to COVID‐19. Meanwhile, the proportion of AA patients increased from 1.02% to 2.71% (*p* = 0.001) and that of TE patients from 0.40% to 2.17% (*p* = 0.001)
6. Turan Ç, et al. *Dermatol Ther*. 2020;33(4).^6^	AA	Evaluation of the diagnostic spectrum in dermatology outpatients indicated that the proportion of patients with AA increased from 1.4% to 2.7% because of COVID‐19 (*p* = 0.017)
7. Turkmen D, et al. *Dermatol Ther*. 2020;33(6).^7^	TE, AA	As per the result of an online questionnaire for individuals who had to stay at home for a long time, TE was seen in 27.9% and AA on the scalp was seen in 2.8% of 563 patients
8. Starace M, et al. *JAAD Int*. 2021;5:11–8.^8^	TE, trichodynia	All 39 patients experienced excessive hair loss within 2–3 months after COVID‐19 disease. Telogen effluvium was observed in 66.3% of patients. The pull tests for telogen effluvium assessment were strongly positive. Fifteen (38.5%) and 24 (61.5%) patients were respectively with mild and moderate COVID‐19 and did not require hospitalization
9. Rossi A, et al. *Skin Appendage Disord*. 2021;21(5):1–5.^9^	TE	As a result of 14 patients' evaluation, TE occurred about 2 months (range, 1–3 months) after SARS‐CoV‐2 infection. The median duration of hair loss was 5 months (range, 1–6 months)
10. Sharquie KE, Jabbar RI. *Ir J Med Sci*. 2021; doi: 10.1007/s11845‐021‐02754‐5.^10^	TE	All 39 patients experienced excessive hair loss within 2–3 months after COVID‐19 disease. The pull tests for TE assessment were strongly positive
11. Rizzetto G, et al. *Dermatol Ther*. 2021;34(1).^11^	TE, AE (identical to the case reported in Ref. 13)	A case report of three TE cases occurring after SARS‐CoV‐2 infection. A case report of one AE case (identical to case in Ref. 13) during severe SARS‐CoV‐2 infection was also mentioned
12. FIvenson D. *Int J Dermatol*. 2021;60(1):127.^12^	AA	Three cases of rapidly progressive AA has been noted in relationship to COVID‐19 possibly due to stress of, quarantine, and/or fear of infection
13. Tanacan E, et al. *Dermatol Ther*. 2020;33(6).^14^	Scarring alopecia	The rate of cicatricial hair loss in the pandemic period was significantly higher compared to that in non‐pandemic period (*p* = 0.009)
14. Turkmen D, et al. *Int J Clin Pract*. 2021;75(4).^15^	AGA	A comparison of 519 men in the non‐pandemic period and 568 men in the pandemic period was conducted; the number of cases of AGA in the pandemic period was low (*p* = 0.0174)
15. Salazar Arenas MÁ, et al. *Infez Med*. 2021;29(1):37–45.^16^	AGA	A cross‐sectional study of 98 male patients diagnosed with COVID‐19 was performed; 45.9% had AGA
16. Almeida G, et al. *J Drugs Dermatol*. 2021;20(1):76–83.^18^	Probable TE?	Forty‐five patients were included in a retrospective chart review, which identified seven major types of cutaneous manifestations. One of them is hair loss (probably TE)
17. Aşkın Ö, et al. *Dermatol Ther*. 2021;34(2).^19^	Other (continued AA treatment)	If the benefits outweigh the risks, patients are recommended to continue tofacitinib therapy for AA during the COVID‐19 pandemic
18. Cadegiani FA, et al. *Cureus*. 2021;13(2).^20^	Other (COVID‐19 treatment)	The addition of an early antiandrogen therapy with dutasteride commonly used for the treatment of AGA can effectively reduce SARS‐CoV‐2 infection in combination with nitazoxanide and azithromycin
19. Gupta AK, et al. *J Cosmet Dermatol*. 2021;20(3):929–36.^21^	Miscellaneous (Google trend search)	A survey of global trends in Google searches for hair loss products (2004–2020) revealed that the most searched term was minoxidil
20. Kutlu Ö. *Dermatol Ther*. 2020;33(6).^22^	Miscellaneous (Google trend search)	As per Google trends, during the COVID‐19 pandemic, the interest in dermatologic terms such as “acne,” “eczema,” and “hair loss” in Turkey and Italy
21. Olds H et al. *Dermatol Ther*. 2021;e14761.^26^	TE	Ten of 522 patients diagnosed with COVID‐19 were diagnosed to have TE caused by COVID‐19

Abbreviations: AA, alopecia areata; AE, anagen effluvium; AGA, androgenetic alopecia; SARS‐CoV‐2, severe acute respiratory syndrome coronavirus 2; TE, telogen effluvium.

Seventeen articles indicated an association between hair loss and COVID‐19 disease, of which three were large cohort studies.^1^, ^2^, ^3^ Hair loss was clearly reported as a sequela of COVID‐19 in all the articles. One systematic review and meta‐analysis^1^ evaluated 47 910 patients from 15 studies and reported that more than 80% of patients had some sequelae after COVID‐19, of which 25% were hair loss.^1^ Another retrospective study of 1946 inpatients with COVID‐19 in the United Kingdom indicated a hair loss frequency of 6%.^2^ Furthermore, a prospective study of 538 patients with a third COVID‐19 infection reported hair loss in 28.6% of patients.^3^

Thirteen of 21 reports specified diseases/conditions that caused hair loss, and seven of 13 reports described TE. The incidence of TE tended to increase during the pandemic.^7^ A survey of 128 COVID‐19‐affected individuals revealed that TE was observed in 66.3% and trichodynia in 58.4% of patients.^8^ In addition, TE was associated with trichodynia in 42.4% of cases.^8^ Furthermore, the results of the hair pull test, trichoscopic investigation, and trichogram of 14 patients who suffered from HLASCI showed typical TE patterns; eight of 14 patients showed a telogen rate of more than 25% as detected by trichogram, and six patients showed a telogen rate of 20% or less tested after over 3 months from hair loss beginning.^9^ In this report, HLASCI cases demonstrated typical trichoscopic findings of TE; regrowing hairs, follicular units with one hair, empty hair follicles, and thin terminal hairs.^9^ In this cohort, TE as HLASCI seemed to occur earlier than classic acute TE. The onset of TE after COVID‐19 reported to be after a median of 2 months (range, 1–3 months) and the median duration 5 months (range, 1–6 months) after infection, while classic acute TE is usually observed 3–4 months after triggering events.^9^ The underlying mechanism of HLASCI was postulated to be exposure to a milieu of inflammatory cytokines or direct viral damage to hair follicles.^9^ In another study, excessive hair loss was reported to have occurred within 2–3 months after infection.^10^ In some patients/cases with TE, hair loss began more than 12 weeks after infection was documented.^8^ Exacerbation of pre‐existing TE could occur in correlation with the stress of lockdown.^11^

Five articles reported AA.^4^, ^5^, ^6^, ^7^, ^12^ Similar to the findings for TE, a statistically significant increase in AA was reported in patients with COVID‐19.^5^, ^6^ Furthermore, not only new onset but also rapid progression and relapse of pre‐existing diseases were reported in COVID‐19 patients with AA.^4^, ^12^ The onset and relapse of AA are speculated to be triggered by a cytokine storm caused by COVID‐19 or the stress associated with being quarantined.^4^, ^12^

For other hair loss clinical subtypes of HLASCI, one case each of anagen effluvium (AE),^11^, ^13^ scarring alopecia^14^ were reported. The case of AE was considered to be a result of anagen hair loss, likely due to profound inflammatory destruction of the hair follicle bulb that occurs abruptly after COVID‐19 infection.^13^ The incidence of scarring alopecia was reported to be statistically significantly higher during the pandemic period than before the pandemic period.^14^ In contrast, a statistically significant decrease in AGA was noted during the pandemic.^15^ On the other hand, a survey of 98 COVID‐19 patients indicated that the severity of COVID‐19 infection was moderate to severe in 13.2% of patients without AGA and in 88.9% of patients with AGA.^16^ This may be due to the increase in transmembrane serine protease 2 (TMPRSS2) and/or angiotensin‐converting enzyme 2 (ACE‐2) by dihydrotestosterone (DHT) binding to the androgen receptor, resulting in the cleavage of SARS‐CoV‐2 virus spike protein, which may allow efficient infection by the virus.^17^ Although further investigation is mandated, AGA might not be exacerbated by COVID‐19 but potentially represents a predisposing or risk factor.

Additional articles less directly described possible association between COVID‐19 and hair loss.^18^, ^19^, ^20^, ^21^, ^22^ Intriguingly, a clinical study suggested that dutasteride, a commonly used medication for AGA, in combination with nitazoxanide and azithromycin could reduce viral shedding and inflammatory markers.^20^ Studies using “Google trend search” suggested growing global interest in hair loss during COVID‐19.^21^, ^22^

### 3.2 telogen effluvium as the main pathomechanism of HLASCI

Based on the present survey, the main pathomechanism of HLASCI can be speculated to be TE. Described above as hair loss due to direct effects of COVID‐19, TE has been known to be associated with various infectious diseases such as typhoid, malaria, human immunodeficiency virus infection, tuberculosis, syphilis, Mediterranean spotted fever, dengue fever, and other febrile illnesses.^23^, ^24^ The observation that HLASCI often developed 1–3 months later is in line with the hypothesis that HLASCI is predominated by TE as the condition usually appears 2–4 months after causative events.^9^, ^25^ Therefore, we conducted a survey to further accumulate evidence on the association of TE with HLASCI.

**FIGURE 1:**
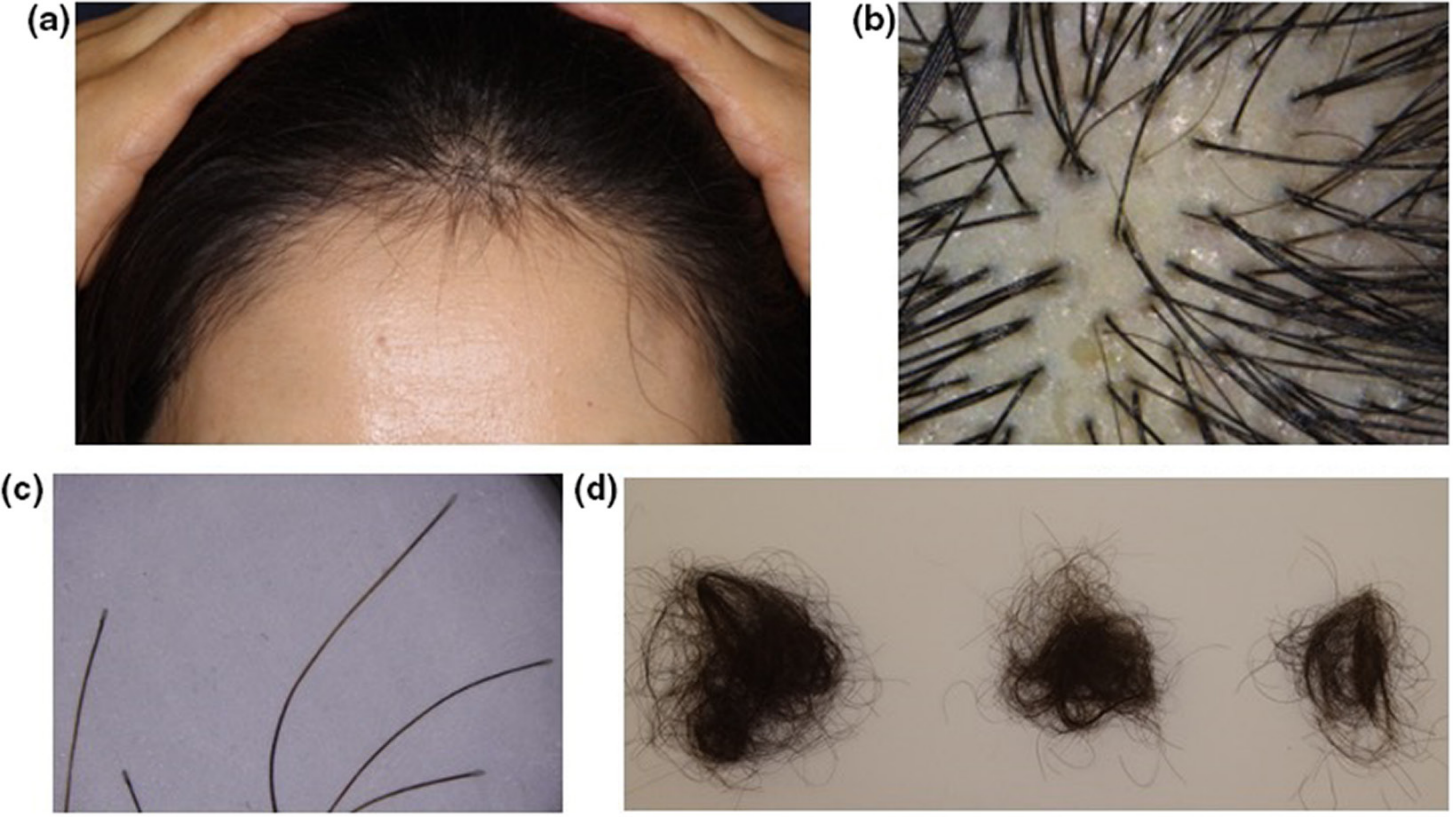
A representative case of HLASCI. A 57‐year‐old female with a two‐month history of progressive hair loss that started approximately 2 months after SARS‐CoV‐2 infection (confirmed by polymerase chain reaction). (a) Decrease in hair density with regrowing hairs observed on the frontal scalp. (b) Upright regrowing hairs and a vacant follicular ostia as observed by trichoscopy. (c) Shed hairs with club‐shaped root. (d) Spontaneous and gradual decrease in hair shedding, typical of telogen effluvium. HLASCI, hair loss after severe acute respiratory syndrome coronavirus 2 infection; SARS‐CoV‐2, severe acute respiratory syndrome coronavirus 2.

The survey was conducted using the search string 2 presented in Table 1. Seven articles of COVID‐19 and TE was extracted using the selected search and inclusion/exclusion criteria and six of which articles were duplicates with search string 1. According to one representative article, ten of 522 patients diagnosed with COVID‐19 were diagnosed to have TE caused by COVID‐19.^26^ On average, hair loss began 50 days after the first manifestation of COVID‐19 infection in these patients.^26^ This report further underscored that COVID‐19 infection is a significant trigger for TE. Figure 1 shows a typical case of HLASCI in which hair loss typical of TE was confirmed approximately 2 months after SARS‐CoV‐2 infection.

### 3.3 Theoretical etiopathogenesis/pathophysiology of HLASCI

**FIGURE 2:**
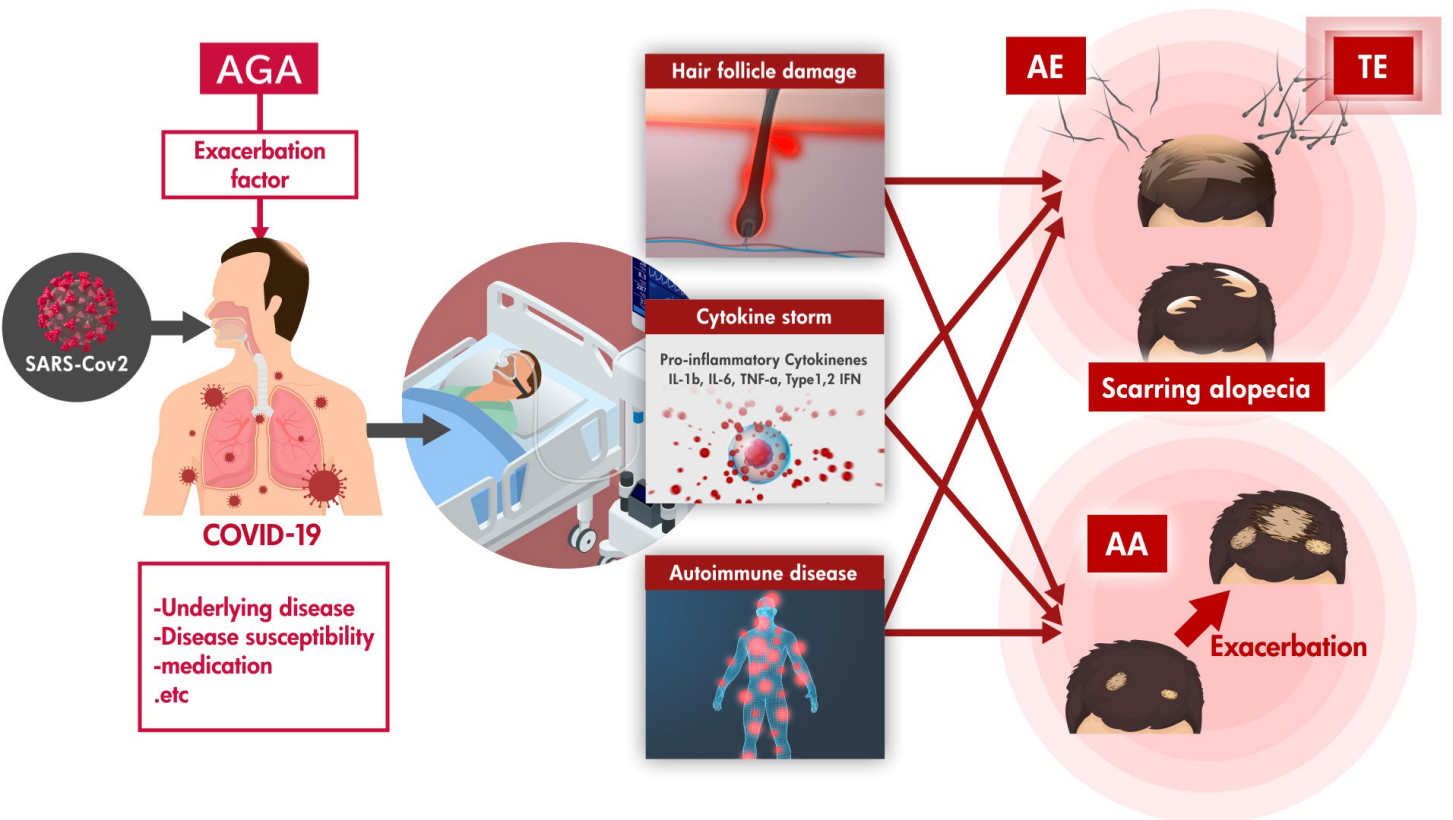
Theoretical explanation of HLASCI etiopathogenesis/pathophysiology. Various factors such as intrinsic susceptibility (e.g. presence of androgenetic alopecia; AGA?), presence of underlying diseases, and medications may predispose individuals to COVID‐19. Infection with SARS‐CoV‐2 results in a cytokine storm. Hair follicles by themselves can be targets of SARS‐CoV‐2. Induced anagen hair follicle damage can lead to telogen effluvium (TE), anagen effluvium and contribute to exacerbation of alopecia areata (AA). HLASCI, hair loss after severe acute respiratory syndrome coronavirus 2 infection; COVID‐19, coronavirus disease 2019; SARS‐CoV‐2, severe acute respiratory syndrome coronavirus 2.

The theoretical etiopathogenesis/pathophysiology of HLASCI inferred from the above outcomes is summarized in Figure 2. Infection with SARS‐CoV‐2 results in increased levels of inflammatory cytokines (e.g., interleukin [IL]‐6, interferon [IFN] alpha‐2b), resulting in a cytokine storm.^9^ Hair follicles enter quiescence due to biological insults such as excessive IFNs, possibly leading to acute TE. Various factors such as intrinsic susceptibility, presence of underlying diseases, and medications may predispose individuals to COVID‐19. AGA may be involved as one of the underlying diseases affecting the susceptibility to infection and severity of COVID‐19.^16^ SARS‐CoV‐2 infection‐induced anagen hair follicle damage can be a factor in the development and recurrence of AA.^4^, ^5^, ^6^, ^7^, ^12^ In addition, other events involving COVID‐19‐infected individuals, such as quarantine, may exacerbate autoimmune disease due to extreme physiological stress, resulting in exacerbation of AA.

### 3.4 Management of HLASCI

It is of paramount importance to control the inflammation caused by COVID‐19 to manage HLASCI. Control of comorbidities and physiological stress may also be essential in reducing the incidence or magnitude of HLASCI.

Given that TE is assumed to be the main cause of HLASCI, a survey of the strategies for improvement of TE would be advantageous in the management of HLASCI. TE is characterized by an increasing ratio of telogen hairs. Under normal conditions, anagen (growing) hair follicles account for 90%–95% of the total follicles on the scalp, with the remaining 5%–10% of hair follicles in the telogen phase.^27^ In TE, the ratio of telogen is reported to increase to more than 25%.^24^, ^27^ TE is classified into three groups: acute TE, chronic TE, and chronic diffuse telogen hair loss.^25^ Acute TE typically presents transient hair loss subsequent to febrile illness, major surgery, or crash diet, among others.^25^ Acute TE is usually expected to spontaneously recover within a couple of months once the causative factors are identified and addressed.^25^ Thus, acute TE as a representative manifestation of HLASCI can be expected to recover approximately 2–3 months after COVID‐19 recovery. Nevertheless, patients' quality of life may be significantly impaired by hair loss due to emotional stress, sometimes hindering their social life. Accordingly, earlier recovery from acute TE is desirable.

An additional survey on TE and treatment using search string 3 identified 11 papers (Table 1).^28^, ^29^, ^30^, ^31^, ^32^, ^33^, ^34^, ^35^, ^36^, ^37^, ^38^ Currently, no established treatments or guidelines on TE treatment are available.^23^ Various studies on treatment have been conducted.^28^, ^29^, ^30^, ^31^, ^32^, ^33^, ^34^, ^35^, ^36^, ^37^, ^38^ The most common treatment was oral minoxidil (Table 3).^28^, ^29^, ^30^ Literature reviews of 17 studies with 634 patients receiving oral minoxidil as the primary treatment for hair loss indicated that oral minoxidil was an effective and well‐tolerated primary medication to treat not only AGA but also other types of alopecia, including TE and AA with only minor adverse events.^28^ Other studies (10 reports) assessing 19 218 patients (215 women and 19 003 men) reported that, in addition to the strongest evidence for the efficacy of oral minoxidil for treating AGA and AA, oral minoxidil was effective in treating female pattern hair loss and chronic TE.^29^ Accordingly, minoxidil may be a therapeutic option for TE.^30^ However, oral minoxidil administration is not recommended as per the Japanese Dermatological Association (JDA) AGA management guideline because of possible adverse events.^39^ A larger randomized controlled trial with more standardized objective measures is essential to optimize treatment protocols, such as dosing and duration, to assess the real risk/benefit balance of oral minoxidil to the treatment of hair loss, including TE.

**TABLE 3:** Articles describing minoxidil treatment for telogen effluvium extracted by string 3

Journal	Type of treatment	Summary of reports	Type of article
Randolph M, Tosti A. *J Am Acad Dermatol*. 2021;84(3):737–46.^28^	Oral minoxidil	Oral minoxidil was found to be an effective and well‐tolerated alternative treatment option for AGA, TE, lichen planopilaris, loose anagen syndrome, monilethrix, AA, and permanent chemotherapy‐induced alopecia	Key word search using PubMed
Sharma AN, et al. *Int J Dermatol*. 2020;59(8):1013–9.^29^	Low doses of oral minoxidil	Based on ten articles (comprising a total 19 218 patients, oral minoxidil (0.25‐5 mg/day~twice daily) achieved clinical improvement in 86.1% of chronic TE cases	PubMed database search in accordance with PRISMA guideline
Villani A, et al. J *Eur Acad Dermatol Venereol*. 2021;35(7):1485–92.^30^	Oral minoxidil	With the use of oral minoxidil in 36 female TE patients, mean hair shedding scores improved both at 6 and 12 months	Data base search in PubMed, The Cochrane Library, Embase, Google Scholar, EBSCO and Scopus

Abbreviations: AA, alopecia areata; AGA, androgenetic alopecia; LED, light emitting diode; TE, telogen effluvium.

**FIGURE 3:**
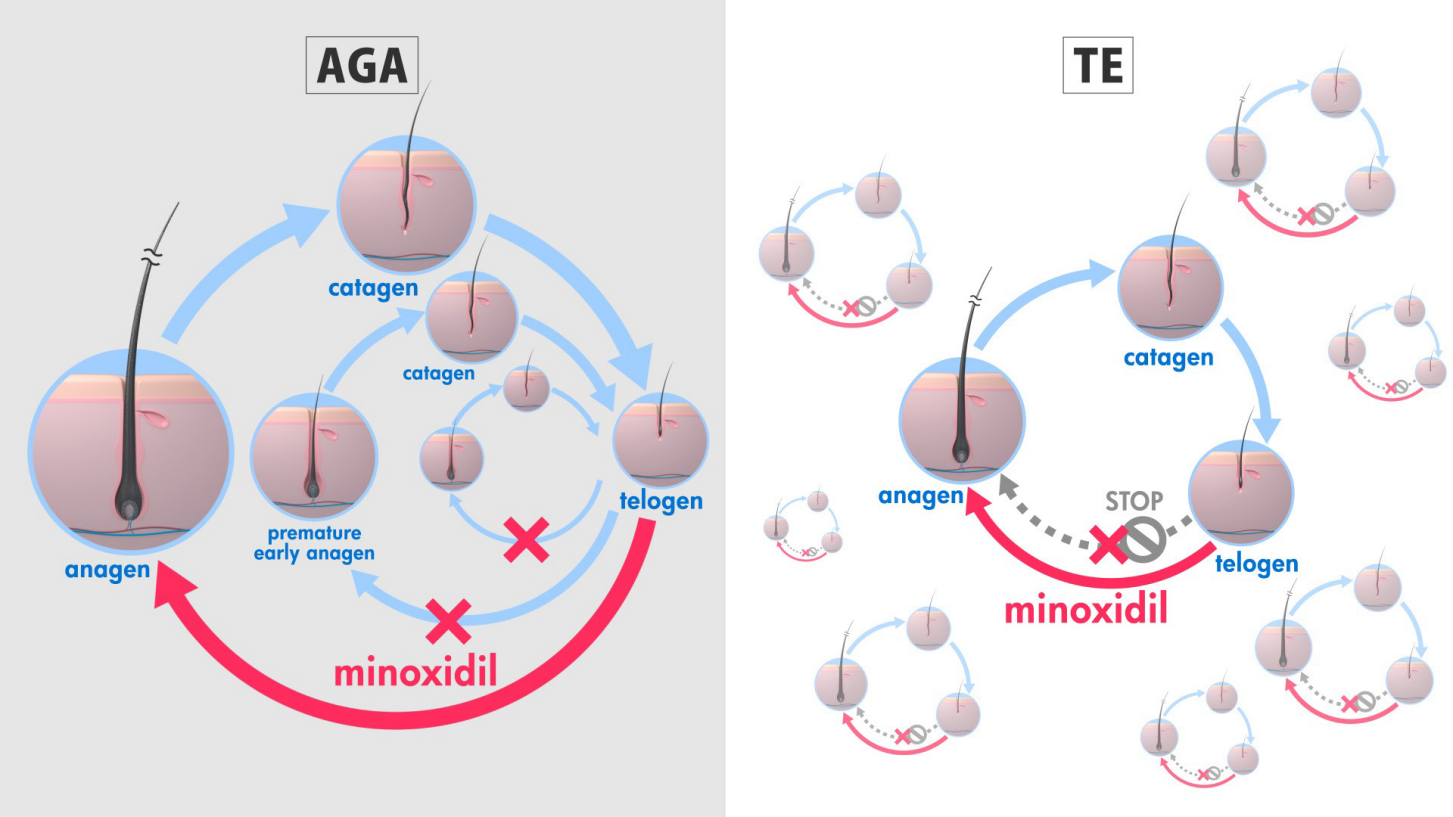
Effect of minoxidil to normalize the abnormal hair cycle in androgenetic alopecia (AGA) and telogen effluvium (TE; for TE hypothetical). In AGA, minoxidil inhibits acceleration of the hair cycle to prevent further hair follicle miniaturization and enhance anagen entry. Analogous anagen initiation can be expected in TE with resultant improvement of hair loss.

Of note, topical minoxidil is widely used in Japan and overseas for AGA treatment and is recommended in the JDA guideline. Within the growth cycle of hair follicles, apoptosis is induced in hair matrix cells when the anagen phase transitions to telogen.^40^ The mode of action of minoxidil to promote hair growth has been speculated to be the activation of sulfonylurea receptors, improvement of hair follicle blood flow by opening vascular smooth muscle ATP‐sensitive K^+^ channels, production of cellular growth factors such as vascular endothelial growth factor from the dermal papilla cells,^41^ and/or the inhibition of dermal papilla cells apoptosis at the time of transition from anagen to catagen/telogen.^42^ Consequently, minoxidil can inhibit shortening of the anagen phase induced by androgens in AGA and promote the shift from the telogen to early anagen phase (Figure 3). Thus, topical minoxidil is expected to be useful for the treatment of TE.

In cases of AGA and HLASCI, hair loss may be more apparent. Use of topical minoxidil would be preferentially supported in such cases. Minoxidil has also been reported to be efficacious for treatment of AA.^35^ Exacerbation of AA has been implicated in the mechanisms of HLASCI,^4^, ^12^ and topical minoxidil would be beneficial in this scenario, especially when primary inflammatory change is reduced.

Besides minoxidil, promise of various therapeutic approaches, including biotin^31^ or iron^32^ supplementation, growth factors vacuolated through iontophoresis,^33^ antioxidant and anti‐inflammatory shampoo and lotion,^34^ vitamin D_3_ intake,^35^ microneedles,^36^ sandalwood,^37^ and photobiostimulation combined with microinjection,^38^ have been explored to treat TE. Other remedies, which was not retrieved by search string 3, including an oral formulation containing l‐cystine, thiamin, calcium d‐pantothenate, medicinal yeast, keratin and p‐aminobenzoic acid, may also be beneficial to improve TE;^43^ however, current evidence levels are relatively low and high‐quality, specific, prospectively designed, randomized controlled trials are essential to establish their efficacy.

## 4 FUTURE DIRECTION

In our attempt to dissect the association between hair loss conditions and COVID‐19, we delineated some links: AGA as a putative risk factor for COVID‐19, exacerbation of pre‐existing AA by COVID‐19, and acute TE after COVID‐19. Acute TE would explain the basis of the majority of HLASCI cases. We are aware that several confounding factors exist in the reported events, and hence, further accumulation of cases is necessary to fully characterize HLASCI. In addition, the duration of this literature survey was from January 2020 to September 2021 when the delta strain was predominant, while the omicron strain with putatively less severe COVID‐19 symptoms was mainstream as of February 2022 (at the time of writing). How the frequency of HLASCI is influenced by this change in strains needs to be evaluated by further accumulation of cases. The search results could be more robust by adopting multiple search strings using different terminologies describing the same or analogous conditions. Recent drastic environmental changes, particularly global warming, may lead to a new pandemic in the future, which can trigger acute TE, as observed in HLASCI. Further study of HLASCI can provide a basis for understanding such conditions.

## 5 CONCLUSION

Although further study of the pathophysiology underlying HLASCI is necessary, this literature review indicates that the main cause of HLASCI is acute TE. Topical minoxidil, as an adjunct to optimal care of patients with COVID‐19 disease, represents a favorable therapeutic option to better manage HLASCI.

## CONFLICT OF INTEREST

M.O. is a medical advisor for Taisho Pharmaceutical Co., Eli Lilly Japan K.K., Pfizer Japan Inc., and ROHTO Pharmaceutical Co. and receives advisory fees. He also receives research grants for projects not related to this study from Maruho Co., Sun Pharma Japan Ltd., and Shiseido Co. T.F. and K.M. are employees of Taisho Pharmaceutical Co.

## References

[1] Lopez‐Leon S , Wegman‐Ostrosky T , Perelman C , Sepulveda R , Rebolledo P , Cuapio A , et al. More than 50 long‐term effects of COVID‐19: a systematic review and meta‐analysis. Res Sq. 2021. 10.21203/rs.3.rs-266574/v1 PMC8352980 34373540

[2] Cheng D , Calderwood C , Skyllberg E , Ainley A . Clinical characteristics and outcomes of adult patients admitted with COVID‐19 in East London: a retrospective cohort analysis. BMJ Open Respir Res. 2021;8:e000813. 10.1136/bmjresp-2020-000813 PMC7976675 33731329

[3] Xiong Q , Xu M , Li J , Liu Y , Zhang J , Xu Y , et al. Clinical sequelae of COVID‐19 survivors in Wuhan, China: a single‐centre longitudinal study. Clin Microbiol Infect. 2021;27:89–95. 32979574 10.1016/j.cmi.2020.09.023 PMC7510771

[4] Rinaldi F , Trink A , Giuliani G , Pinto D . Italian survey for the evaluation of the effects of coronavirus disease 2019 (COVID‐19) pandemic on alopecia areata recurrence. Dermatol Ther (Heidelb). 2021;11:339–45. 33580408 10.1007/s13555-021-00498-9 PMC7880634

[5] Kutlu Ö , Metin A . Relative changes in the pattern of diseases presenting in dermatology outpatient clinic in the era of the COVID‐19 pandemic. Dermatol Ther. 2020;33:e14096. 32869938 10.1111/dth.14096

[6] Turan Ç , Metin N , Utlu Z , Öner Ü , Kotan ÖS . Change of the diagnostic distribution in applicants to dermatology after COVID‐19 pandemic: what it whispers to us? Dermatol Ther. 2020;33:e13804. 32530503 10.1111/dth.13804 PMC7300472

[7] Turkmen D , Altunisik N , Sener S , Colak C . Evaluation of the effects of COVID‐19 pandemic on 336 hair diseases through a web‐based questionnaire. Dermatol Ther. 2020;33:e13923. 32594627 10.1111/dth.13923 PMC7361059

[8] Starace M , Iorizzo M , Sechi A , Alessandrini AM , Carpanese M , Bruni F , et al. Trichodynia and telogen effluvium in COVID‐19 patients: results of an international expert opinion survey on diagnosis and management. JAAD Int. 2021;5:11–8. 34368790 10.1016/j.jdin.2021.07.006 PMC8328568

[9] Rossi A , Magri F , Sernicola A , Michelini S , Caro G , Muscianese M , et al. Telogen effluvium after SARS‐CoV‐2 infection: a series of cases and possible pathogenetic mechanisms. Skin Appendage Disord. 2021;7:377–81. 10.1159/000517223 PMC8339054 34373830

[10] Sharquie KE , Jabbar RI. COVID‐19 infection is a major cause of acute telogen effluvium. Ir J Med Sci. 2021. 10.1007/s11845-021-02754-5 PMC8407603 34467470

[11] Rizzetto G , Diotallevi F , Campanati A , Radi G , Bianchelli T , Molinelli E , et al. Telogen effluvium related to post severe Sars‐Cov‐2 infection: clinical aspects and our management experience. Dermatol Ther. 2021;34:e14547. 33190397 10.1111/dth.14547 PMC7744849

[12] FIvenson D . COVID‐19: association with rapidly progressive forms of alopecia areata. Int J Dermatol. 2021;60:127. 33226118 10.1111/ijd.15317 PMC7753616

[13] Shanshal M . COVID‐19 related anagen effluvium. J Dermatolog Treat. 2020;33:1114–5. 32628051 10.1080/09546634.2020.1792400

[14] Tanacan E , Aksoy Sarac G , Emeksiz MAC , Dincer Rota D , Erdogan FG . Changing trends in dermatology practice during COVID‐19 pandemic: a single tertiary center experience. Dermatol Ther. 2020;33:e14136. 32767466 10.1111/dth.14136 PMC7435568

[15] Turkmen D , Altunisik N , Mantar I , Durmaz I , Sener S , Colak C . Comparison of patients' diagnoses in a dermatology outpatient clinic during the COVID‐19 pandemic period and pre‐pandemic period. Int J Clin Pract. 2021;75:e13948. 33332694 10.1111/ijcp.13948 PMC7883201

[16] Salazar Arenas MA , Carpio‐Toia AMD , Galdos JA , Rodriguez‐Morales AJ . Alopecia and severity of COVID‐19: a cross‐sectional study in Peru. Infez Med. 2021;29:37–45. 33664171

[17] Moradi F , Enjezab B , Ghadiri‐Anari A . The role of androgens in COVID‐19. Diabetes Metab Syndr. 2020;14:2003–6. 33091758 10.1016/j.dsx.2020.10.014 PMC7557269

[18] Almeida G , Arruda S , Marques E , Michalany N , Sadick N . Presentation and management of cutaneous manifestations of COVID‐19. J Drugs Dermatol. 2021;20:76–83. 33400417 10.36849/JDD.5676

[19] Aşkın Ö , Özkoca D , Uzunçakmak TK , Serdaroğlu S . Evaluation of the alopecia areata patients on tofacitinib treatment during the COVID‐19 pandemic. Dermatol Ther. 2021;34:e14746. 33405372 10.1111/dth.14746 PMC7883269

[20] Cadegiani FA , McCoy J , Wambier CG , Goren A . Early antiandrogen therapy with dutasteride reduces viral shedding, inflammatory responses, and time‐to‐remission in males with COVID‐19: a randomized, double‐blind, placebo‐controlled interventional trial (EAT‐DUTA AndroCoV trial ‐ biochemical). Cureus. 2021;13:e13047. 33643746 10.7759/cureus.13047 PMC7885746

[21] Gupta AK , Quinlan EM , Williams KL . The shifting preferences of patients and physicians in nonsurgical hair loss treatment. J Cosmet Dermatol. 2021;20:929–36. 32892459 10.1111/jocd.13681

[22] Kutlu Ö . Analysis of dermatologic conditions in Turkey and Italy by using Google trends analysis in the era of the COVID‐19 pandemic. Dermatol Ther. 2020;33:e13949. 32614116 10.1111/dth.13949 PMC7361070

[23] Chien Yin GO , Siong‐See JL , Wang ECE . Telogen effluvium ‐ a review of the science and current obstacles. J Dermatol Sci. 2021;101:156–63. 33541773 10.1016/j.jdermsci.2021.01.007

[24] Liyanage D , Sinclair R . Telogen effluvium. Cosmetics. 2016;3:13.

[25] Grover C , Khurana A . Telogen effluvium. Indian J Dermatol Venereol Leprol. 2013;79:591–603. 23974577 10.4103/0378-6323.116731

[26] Olds H , Liu J , Luk K , Lim HW , Ozog D , Rambhatla PV . Telogen effluvium associated with COVID‐19 infection. Dermatol Ther. 2021;34:e14761. 33405302 10.1111/dth.14761 PMC7883200

[27] Malkud S . Telogen effluvium: a review. J Clin Diagn Res. 2015;9:WE01–3. 26500992 10.7860/JCDR/2015/15219.6492 PMC4606321

[28] Randolph M , Tosti A . Oral minoxidil treatment for hair loss: a review of efficacy and safety. J Am Acad Dermatol. 2021;84:737–46. 32622136 10.1016/j.jaad.2020.06.1009

[29] Sharma AN , Michelle L , Juhasz M , Ramos PM , Mesinkovska NA . Low‐dose oral minoxidil as treatment for non‐scarring alopecia: a systematic review. Int J Dermatol. 2020;59:1013–9. 32516434 10.1111/ijd.14933

[30] Villani A , Fabbrocini G , Ocampo‐Candiani J , Ruggiero A , Ocampo‐Garza SS . Review of oral minoxidil as treatment of hair disorders: in search of the perfect dose. J Eur Acad Dermatol Venereol. 2021;35:1485–92. 33660357 10.1111/jdv.17216

[31] Abdel Rahman SH , Salem RM , Sabry JH . Biotin deficiency in telogen effluvium: fact or fiction? J Clin Aesthet Dermatol. 2020;13:37–40. PMC7159307 32308796

[32] Yorulmaz A , Hayran Y , Ozdemir AK , Sen O , Genc I , Aksoy GG . Telogen effluvium in daily practice: patient characteristics, laboratory parameters, and treatment modalities of 3028 patients with telogen effluvium. J Cosmet Dermatol. 2021. 10.1111/jocd.14413 34449961

[33] Alessandrini AM , Bruni F , Piraccini MB , Starace M . The effectiveness and tolerability of preformed growth factors vehiculated through iontophoresis on patients with androgenetic alopecia and telogen effluvium: a clinical study. Dermatol Pract Concept. 2021;11:e2021082. 34123571 10.5826/dpc.1103a82 PMC8172030

[34] Anzai A , Pereira AF , Malaquias KR , Guerra LO , Mercuri M . Efficacy and safety of a new formulation kit (shampoo + lotion) containing anti‐inflammatory and antioxidant agents to treat hair loss. Dermatol Ther. 2020;33:e13293. 32134172 10.1111/dth.13293

[35] Sattar F , Almas U , Ibrahim NA , Akhtar A , Shazad MK , Akram S , et al. Efficacy of oral vitamin D_3_ therapy in patients suffering from diffuse hair loss (telogen effluvium). J Nutr Sci Vitaminol (Tokyo). 2021;67:68–71. 33642467 10.3177/jnsv.67.68

[36] Starace M , Alessandrini A , Brandi N , Piraccini BM . Preliminary results of the use of scalp microneedling in different types of alopecia. J Cosmet Dermatol. 2020;19:646–50. 31254437 10.1111/jocd.13061

[37] Jimenez F , López E , Bertolini M , Alam M , Chéret J , Westgate G , et al. Topical odorant application of the specific olfactory receptor OR2AT4 agonist, Sandalore®, improves telogen effluvium‐associated parameters. J Cosmet Dermatol. 2021;20:784–91. 32645251 10.1111/jocd.13608

[38] Piccolo D , Crisman G , Conforti C , Buzzi M , Genovesi C , Marchi D , et al. Trichobiolight: a new, effective protocol in the treatment of androgenetic alopecia and telogen effluvium. Dermatol Ther. 2021;34:e14799. 33486860 10.1111/dth.14799

[39] Manabe M , Tsuboi R , Itami S , Osada S‐I , Amoh Y , Ito T , et al. Guidelines for the diagnosis and treatment of male‐pattern and female‐pattern hair loss, 2017 version. J Dermatol. 2018;45:1031–43. 29863806 10.1111/1346-8138.14470

[40] Botchkareva NV , Ahluwalia G , Shander D . Apoptosis in the hair follicle. J Invest Dermatol. 2006;126:258–64. 16418734 10.1038/sj.jid.5700007

[41] Messenger AG , Rundegren J . Minoxidil: mechanisms of action on hair growth. Br J Dermatol. 2004;150:186–94. 14996087 10.1111/j.1365-2133.2004.05785.x

[42] Han JH , Kwon OS , Chung JH , Cho KH , Eun HC , Kim KH . Effect of minoxidil on proliferation and apoptosis in dermal papilla cells of human hair follicle. J Dermatol Sci. 2004;34:91–8. 15033191 10.1016/j.jdermsci.2004.01.002

[43] Lengg N , Heidecker B , Seifert B , Trüeb RM . Dietary supplement increases anagen hair rate in women with telogen effluvium: results of a double‐blind, placebo‐controlled trial. Therapy. 2007;4:59–65.

